# Morphological and molecular evidence of the Antarctic sleeper shark *Somniosus antarcticus* (Somniosidae) in northern Chile

**DOI:** 10.7717/peerj.21381

**Published:** 2026-06-26

**Authors:** Angel Mancilla, Leandro Brizuela, Krishna Tapia, Carolina Vargas-Caro, Carlos Bustamante

**Affiliations:** 1 Programa de Conservación de Tiburones, Universidad de Antofagasta, Antofagasta, Chile; 2 CHALLWA, Laboratorio de Biología Pesquera, Instituto de Ciencias Naturales Alexander von Humboldt, Universidad de Antofagasta, Antofagasta, Chile

**Keywords:** *Somniosus pacificus*, Southeastern Pacific, Range extension, COI barcoding, Deep-sea bycatch

## Abstract

Sleeper sharks (family Somniosidae) are large, slow-growing deep-sea sharks that are rarely documented in the southeastern Pacific, and records from the Chilean waters have remained taxonomically uncertain. Here, we report a confirmed record of a large-bodied southern *Somniosus* from northern Chile based on a gravid female incidentally captured by a commercial deep-sea longline fishery off Antofagasta and a late-stage male embryo aborted during handling. The embryo measured 90.5 cm in total length and exhibited morphological and meristic characters consistent with large-bodied *S. antarcticus*, including a cylindrical body, short rounded snout, spineless dorsal fins, hook-like dermal denticles, and a 40-turn spiral valve. Additionally, a 639 bp fragment of the mitochondrial cytochrome C oxidase subunit I (COI) gene was obtained from both the female and embryo, and the two sequences were identical, consistent with maternal inheritance. Comparative analyses using public records placed the Chilean haplotype within the large-bodied *Somniosus* species complex. The Chilean haplotype was identical to an Antarctic record labelled *S. pacificus* and differed by two substitutions from a New Zealand record labelled *S. antarcticus*. However, the COI does not fully resolve species boundaries within the *pacificus*/*antarcticus* complex, as the divergence between nominal species remains low. These results provide the first well-documented record of the Antarctic sleeper shark in northern Chile and expand the documented occurrence of southern large-bodied *Somniosus* along the Chilean margin. More broadly, this record highlights the importance of integrating internal meristics, morphology, and molecular data when documenting rare deep-sea sharks in fishery bycatch.

## Introduction

Sleeper sharks of the genus *Somniosus* Lesueur 1818 are large, slow-growing, deep-sea sharks characterized by the absence of dorsal fin spines, a heterocercal caudal fin with an elongated upper lobe, and a first dorsal fin originating posterior to the pectoral fin bases (Compagno, 1984; Ebert, Fowler & Compagno, 2021). Adults range from approximately 70 cm in the smallest species to >four m in large-bodied forms and inhabit continental and insular slopes, oceanic plateaus, and seamounts from upper slope depths to >2,000 m in polar, temperate, and some tropical regions (Yano, Stevens & Compagno, 2004; Ebert, Fowler & Compagno, 2021).

Yano, Stevens & Compagno (2004) reviewed the systematics of *Somniosus* and recognized two subgenera based on body size, dermal denticles, spiral valve counts, and vertebral calcification: the large-bodied subgenus *Somniosus*, including *S. microcephalus* (North Atlantic and Arctic), *S. pacificus* (North Pacific and adjacent Arctic), and *S. antarcticus* (Southern Hemisphere); and the small-bodied subgenus *Rhinoscymnus*, including *S. longus* and *S. rostratus*. A sixth species, *S. cheni*, was later described in Taiwan within *Rhinoscymnus*, further underscoring the hidden diversity and taxonomic complexity of this group (Hsu, Lin & Joung, 2020).

Historically, large sleeper sharks from the Southern Hemisphere were frequently referred to as *S. microcephalus* or *S. pacificus*, and the validity of *S. antarcticus* has been questioned (*e.g.*, Bass, 1976; Compagno, 1984). Using extensive morphometric and meristic datasets, Yano, Stevens & Compagno (2004) argued that large-bodied southern *Somniosus* are diagnosable as *S. antarcticus*, with proposed differences relative to *S. microcephalus* and *S. pacificus* including dorsal-fin position and height, prebranchial length, spiral valve counts, dentition, and other internal features. Subsequent biological studies have reported that *S. antarcticus* can reach at least 4.5–6.0 m in total length and suggested very late maturation in females (≈4.3–4.5 m in females) and a birth size of ∼40 cm (Yano, Stevens & Compagno, 2007). However, the extent to which these nominal taxa represent discrete evolutionary lineages remains an open question.

Mitochondrial markers have not fully resolved species boundaries within the large-bodied *Somniosus* complex in the past. Barcoding and mitogenomic studies often recover shallow divergence and overlapping haplotypes between *S. pacificus* and *S. antarcticus*, with some Southern Ocean specimens assigned to one species based on morphological clustering closely with the other species genetically (Benz et al., 2007; Murray et al., 2008; Christensen, 2022; Claassens et al., 2023). Recently, phylogenomic evidence has further highlighted the limited differentiation between *S. pacificus* and *S. antarcticus*, raising the possibility that these nominal species may represent a single lineage or an unresolved species complex (Timm et al., 2023). More broadly, limitations in species-level discrimination using COI have also been documented in other elasmobranch datasets, where low divergence among closely related taxa, ambiguity in reference sequences, and conflicts between morphology and barcoding can complicate species assignment, reinforcing the value of integrative approaches (Wong, Shivji & Hanner, 2009; Amini et al., 2025; Khudamrongsawat et al., 2025).

Recent observations and range extensions documented by deep-sea cameras and ROVs have often been conservatively reported as *Somniosus* cf. *pacificus* or *Somniosus* sp. (Acero et al., 2018; Claassens et al., 2023), emphasizing the need for integrative morphological and molecular datasets explicitly linked to well-documented specimens and reproducible reference sequence assemblies. Records of *Somniosus* from the southeastern Pacific, particularly from the waters off Chile, are scarce and taxonomically uncertain. The first documented Chilean specimen was reported as *Somniosus* cf. *pacificus* off Valdivia (39°45′S), based on an adult female captured by a deep-sea fishery (Pequeño, Lamilla & Crovetto, 1991). A second record of *S. pacificus* was reported from San Antonio (33°35′S) in central Chile, based on a stranded male (Brito, 2004). Because these identifications predate the systematic revision of Yano, Stevens & Compagno (2004) and are not supported by genetic data, some Southern Hemisphere records attributed to *S. pacificus* may instead correspond to *S. antarcticus* or, more broadly, to a southern large-bodied *Somniosus* lineage within the *pacificus/antarcticus* complex (Yano, Stevens & Compagno, 2004; Christensen, 2022; Timm et al., 2023).

The southern sleeper shark (*S. antarcticus sensu* Yano, Stevens & Compagno, 2004) is currently assessed as “Least Concern” on the IUCN Red List; however, population trends are largely unknown, and the species is considered naturally uncommon throughout much of its reported range (Finucci, 2018). It is primarily caught as bycatch in deep-sea longline and gillnet fisheries across its distribution range (Ebert & Mostarda, 2016; Ebert, Fowler & Compagno, 2021). In Chile, deep-sea fisheries for teleosts and invertebrates operate along the continental slope and could interact with sleeper sharks; however, verified records of these interactions and specimens suitable for detailed taxonomic appraisal are rare. Here, we report the occurrence of a large-bodied southern *Somniosus* in northern Chile, based on a gravid female captured off the coast of Antofagasta and a late-stage male embryo that was aborted during handling. We (1) provide detailed morphometric and meristic information for the embryo, including dentition and spiral valve counts, and (2) generate COI sequences from the female and embryo to assess the placement of the Chilean haplotype within a comparative public dataset of *Somniosus* species. However, because COI does not fully resolve species boundaries within the *S. pacificus/antarcticus* complex (Timm et al., 2023), we interpret the molecular evidence as supporting a lineage affinity rather than species delimitation. We discuss the implications of this record for documenting large-bodied *Somniosus* in the southeastern Pacific and for improving understanding of deep-sea shark bycatch and conservation in northern Chile.

**Figure 1:**
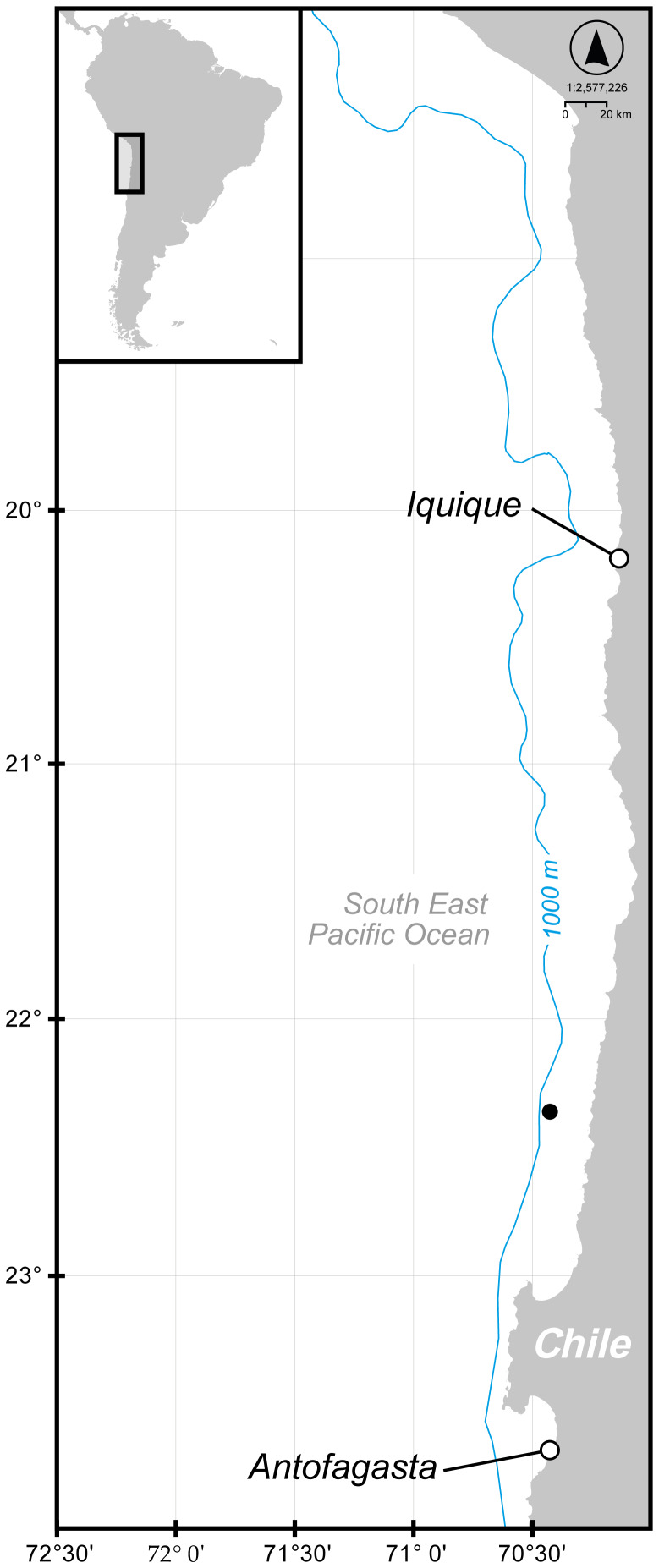
Map of collection site. Geographic location of *Somniosus antarcticus* specimens captured off Antofagasta, northern Chile. The black circle indicates the capture site on the continental slope at ∼22°152S, 70°452W.

## Materials & Methods

### Sampling and specimen preservation

In August 2023, a large female sleeper shark was incidentally captured by a commercial deep-sea longline fishery operating off Antofagasta, northern Chile (22°15′S, 70°45′W) at a depth of approximately 1,000 m depth (Fig. 1). During handling while the female was being lifted onboard, a single small male embryo was expelled and recovered by the fishers (capture-induced abortion). However, owing to operational constraints and the large body size of the female, the specimen could not be retained completely. Only the caudal fin was retained and preserved for documentation and genetic analyses. The embryo was collected whole and fixed in formalin for taxonomic examination. An umbilical opening/scar was visible and used, together with the capture context, to classify the specimen as a late-stage (near-term) embryo. Sex was determined based on the presence of claspers. The embryo was catalogued as CHALLWA-UA-25 and deposited in the ichthyological collection of the Laboratorio de Biología Pesquera (CHALLWA), Universidad de Antofagasta.

### Morphological examination

Morphological and meristic characters were examined following Yano, Stevens & Compagno (2004), with additional comparative information from Ebert & Mostarda (2016). A total of 64 morphometric measurements were obtained for the embryo using a measuring board and calipers, including natural total length (TL), head proportions, fin origins and dimensions, and trunk and caudal measurements. Measurements are reported as percentages of TL and interpreted in the context of published ranges for large-bodied *Somniosus* (Yano, Stevens & Compagno, 2004), recognizing the potential ontogenetic and allometric variation. External characters were documented, including overall body shape, coloration, relative dorsal fin size and position, pectoral and pelvic fin morphology, caudal peduncle shape, and caudal fin proportions. Because only the caudal fin was retained from the female, TL could not be measured directly. However, an approximate estimate of female TL was estimated using a proportional relationship between the upper caudal lobe length and TL (Yano, Stevens & Compagno, 2007). Dentition was examined from the jaws, and tooth counts were recorded for both the upper and lower jaws in the functional series, following Yano, Stevens & Compagno (2004). Dermal denticles were examined under a stereomicroscope using a skin sample taken from the lateral trunk below the first dorsal fin; crown shape and cusp morphology were described, noting that denticle morphology may vary with body region and ontogenetic stage. The body cavity of the embryo was opened to expose the internal organs. The spiral valve was removed, and the total number of turns was counted as a diagnostic characteristic; this value was compared with the published ranges for large-bodied *Somniosus* species (Yano, Stevens & Compagno, 2004; Ebert & Mostarda, 2016).

### Molecular analysis

Genomic DNA was extracted from a small sample of embryo muscle collected prior to fixation and from frozen caudal fin tissue of the female using a standard phenol–chloroform protocol (Sambrook & Russell, 2001). A fragment of the mitochondrial cytochrome c oxidase subunit I (COI) gene was amplified by polymerase chain reaction (PCR) using universal fish barcoding primers, as described by Ward et al. (2005). Amplicons were purified and Sanger sequenced in both directions at the Austral-Omics Sequencing Facility (Valdivia, Chile) using standard protocols. Chromatograms were manually inspected for base-calling errors, and ambiguous sites were verified by comparing forward and reverse reads. Primer regions were trimmed, and a consensus sequence was assembled for each specimen. Female and embryo COI sequences were compared to document the mitochondrial haplotype identity and taxonomic affinity. The identity from the COI analysis was not used to infer kinship; shared haplotypes were reported as consistent with maternal inheritance, whereas the mother–embryo relationship was supported by field observations of capture-induced abortion during handling.

Taxonomic identification was conducted using the NCBI BLAST tool against the GenBank nucleotide database. To contextualize the new sequences, a comparative COI dataset was assembled from GenBank by retaining *Somniosus* records explicitly linked to peer-reviewed publications and/or curated museum specimens. The final dataset included 24 sequences of *Somniosus* spp. and three somniosid sequences representing other genera used as outgroups (Table S1). Sequences were aligned using MUSCLE in Geneious Prime (version 2025.1.1), trimmed to a final length of 639 bp, and used to estimate pairwise genetic distances using the Kimura two-parameter (K2P) model in MEGA v7 (Kumar, Stecher & Tamura, 2016). A median-joining (MJ) haplotype network (Bandelt, Forster & Röhl, 1999) was constructed using NETWORK v10.2.0.0, with epsilon set to zero. Nodes represent haplotypes (node size proportional to haplotype frequency), branches represent mutational steps, and inferred median vectors are represented by small nodes. For clustering visualization, a distance-based neighbor-joining (NJ) tree was reconstructed using Geneious Prime from the K2P distance matrix, with node support assessed using 1,000 bootstrap replicates. The new COI sequences were deposited in GenBank under accession numbers PX559966 –PX559967.

### Ethics and permits

This study did not involve animal experiments or intentional harm. Specimens were obtained as bycatch from vessels targeting Patagonian toothfish (*Dissostichus eleginoides*) and processed in accordance with Chilean regulations. All work was conducted under special research permits granted by the Chilean Subsecretaría de Pesca y Acuicultura (E-PINV-2021-598).

## Results

### Morphological examination

**Figure 2:**
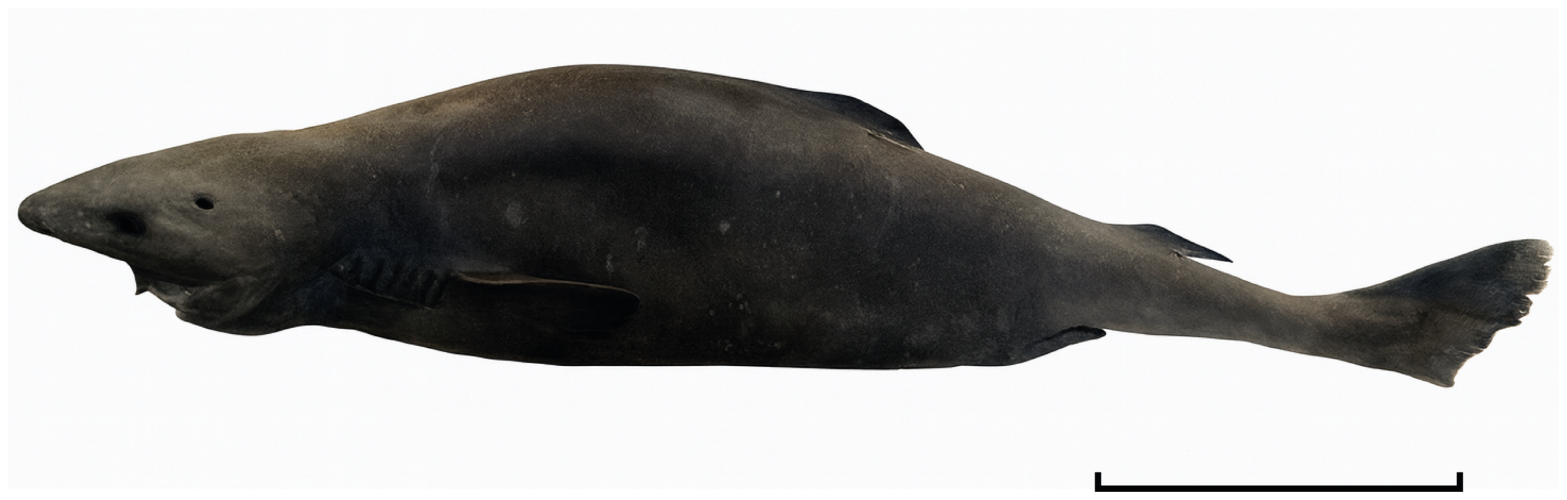
Near-term specimen of *S. antarcticus*. Left lateral view of a near-term male embryo of *Somniosus antarcticus* (CHALLWA-UA-25) specimen examined for morphometric analysis. Scale bar = 25 cm.

The male embryo measured 90.5 cm TL (Fig. 2). Despite some tissue deformation due to fixation, most external and internal characteristics remained intact and could be reliably assessed. The body was cylindrical and uniformly gray. The snout was short and rounded, the eyes were small, and the spiracles were immediately posterior to the eyes. Two spineless dorsal fins of similar size were present; the first dorsal fin originated well behind the pectoral fin bases, and the second dorsal fin originated over the posterior pelvic fin bases. The pectoral and pelvic fins were relatively small. The caudal peduncle lacked a lateral keel, and the caudal fin was asymmetrical, with a markedly longer dorsal than ventral lobe. The small male was recovered after being aborted by the female during handling, confirming female sexual maturity. An umbilical opening/scar was visible, consistent with a late-stage embryo.

**Figure 3:**
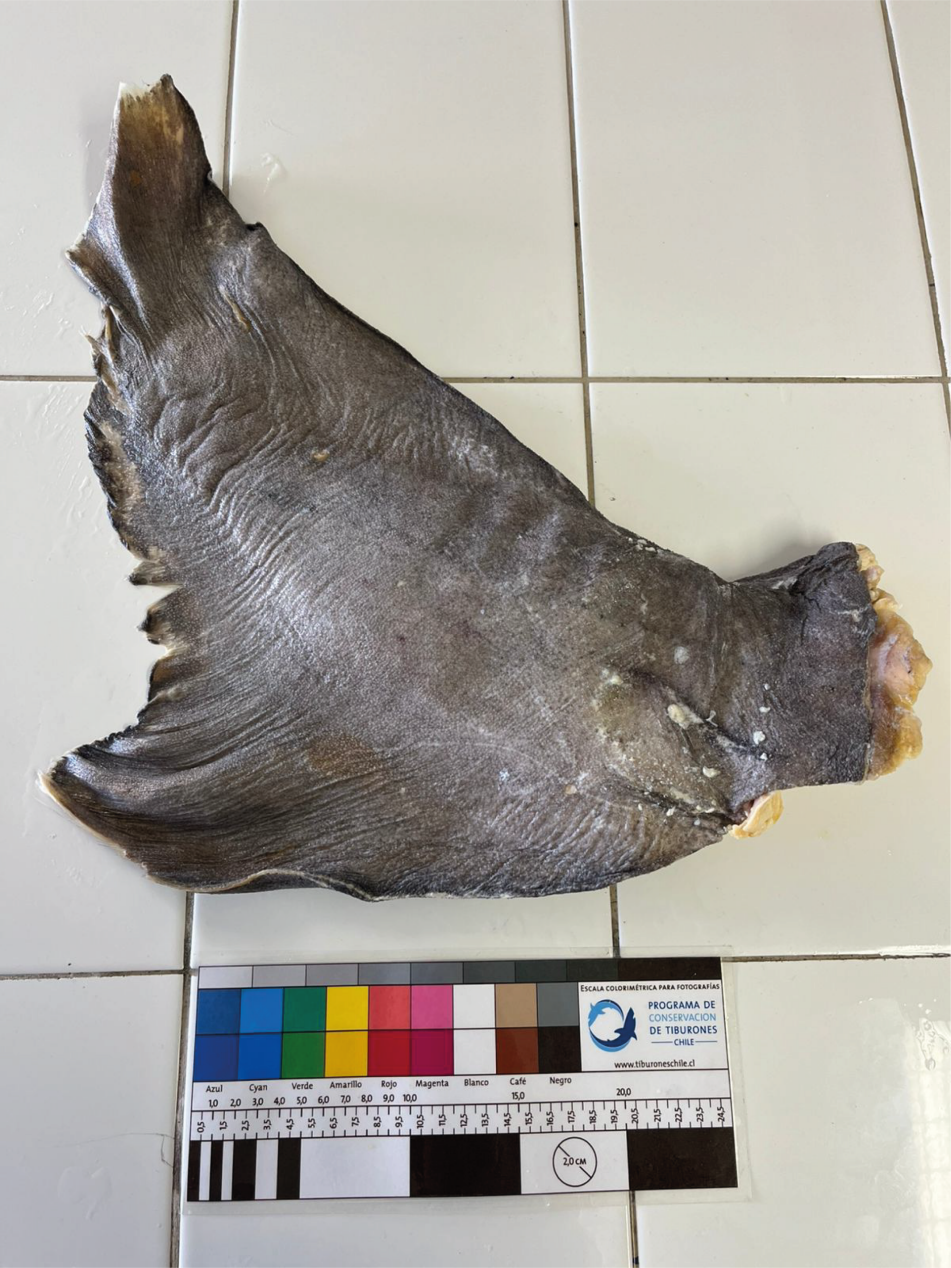
Caudal fin of female specimen of *S. antarcticus*. Lateral view of the caudal fin of an adult female *Somniosus antarcticus* captured off Antofagasta, showing the upper caudal lobe used to estimate the total length. Scale bar = 25 cm.

Morphometric measurements expressed as percentages of TL are presented in Table 1. Values generally overlapped with the ranges reported for large-bodied southern *Somniosus* by Yano, Stevens & Compagno (2004), although some proportional characters are known to vary with ontogeny and among specimens. The upper caudal lobe of the adult female measured 47.5 cm (Fig. 3). Using published proportions for large-bodied southern *Somniosus* (Yano, Stevens & Compagno, 2007), female TL was estimated to fall between approximately 2.2 and 3.0 m. This estimate should be interpreted cautiously, given that it was derived from partial remains.

**Table 1:** Morphometry of *S. antarcticus* from Chilean waters. Morphometric and meristic measurements of the Chilean male embryo (CHALLWA-UA-25), compared with published ranges for large-bodied *Somniosus* species from Yano, Stevens & Compagno (2004). Morphometric values are expressed as percentages of total length (TL), except total length (cm) and meristic characters. Comparative values for *S. microcephalus*, *S. pacificus*, and *S. antarcticus* are shown as minimum–maximum ranges.

**Character**	**CHALLWA-UA-25**	***S. microcephalus***	***S. pacificus***	***S. antarcticus***
Total length (cm)	90.5	64.8–480.0	41.8–430.0	94.0–438.0
**HEAD**				
Snout tip to outer nostrils	3.4	1.3–3.6	1.3–4.4	0.9–3.6
Snout tip to eye	8.2	5.0–8.7	5.5–9.0	4.3–8.9
Snout tip to spiracle	11.7	9.0–13.8	10.0–14.2	9.1–15.2
Snout tip to mouth	9.3	5.4–9.9	4.9–14.0	6.7–12.2
Snout tip to 1st gill opening	17.5	15.1–22.5	15.1–24.2	17.0–23.7
Distance between inner nostril corners	3.3	3.0–4.9	3.3–8.2	3.6–5.1
Interorbital width	9.9	8.7–14.2	8.2–13.1	6.3–12.5
Mouth width	8.1	7.9–10.9	6.5–11.3	7.2–13.9
Eye horizontal diameter	1.9	1.5–3.1	1.2–2.6	0.8–2.1
Eye vertical diameter	1.7	0.6–2.1	0.7–1.8	0.8–1.6
**PREBRANCHIAL AND TRUNK**				
Snout tip to 2nd gill opening	18.9	17.7–22.3	24.2	20.8–25.9
Snout tip to 3rd gill opening	20.3	17.9–25.9	23.2–27.3	22.0–27.0
Snout tip to 4th gill opening	21.0	20.4–26.5	26.8	22.9–28.1
Snout tip to 5th gill opening	22.5	20.0–28.6	25.3–30.8	23.9–30.5
Trunk width at pectoral origin	10.44	10.4–18.7	10.9–20.8	12.1–18.8
Trunk height at pectoral origin	5.0	6.4–20.0	2.1–20.5	9.9–21.7
Snout tip to pectoral fin tip	33.8	34.2–41.1	*	31.0–39.7
Distance between preoral clefts	9.72	5.1–8.1	6.3–13.2	5.4
Eye to 1st gill opening	10.1	9.0–12.9	5.8–13.4	10.6–10.9
**PECTORAL FIN**				
Base length	4.2	4.5–8.2	5.1–9.2	4.8–7.4
Anterior margin	10.4	8.7–13.7	9.9–13.4	9.0–12.6
Distal margin	3.9	3.1–7.4	3.1–7.0	2.8–5.2
Posterior margin	2.7	4.2–8.5	4.0–8.2	4.0–8.4
PELVIC FIN				
Overall length	4.5	7.6–11.0	7.9–10.5	8.0–10.6
Base length	3.6	4.7–7.7	4.1–7.4	4.9–7.5
Anterior margin	5.6	4.3–7.1	4.3–7.1	2.6–7.2
Distal margin	1.2	0.8–5.0	1.9–4.6	1.0–4.7
Clasper length	3.8	3.7–8.4	3.5–5.1	3.7–7.0
Clasper length from pelvic axil	0.5	0.8–2.4	1.0	0.7–1.9
**FIRST DORSAL FIN**				
Origin (AO)^†^	39.7	17.2–36.6	28.8–42.9	27.1–40.6
Origin (PO)^†^	42.5	35.5–45.9	44.2–52.6	45.0–53.1
Overall length (from AO)^†^	19.2	12.7–32.3	16.0–29.4	12.5–30.6
Overall length (from PO)^†^	10.8	9.1–20.9	4.9–13.8	8.7–14.0
Posterior margin	5.2	3.7–6.8	4.4–6.6	3.8–6.9
Height	2.5	2.4–4.5	2.2–3.4	1.9–3.3
**SECOND DORSAL FIN**				
Overall length	8.9	6.6–12.7	9.2–12.8	8.5–24.6
Base length	4.4	5.4–8.8	4.7–7.8	4.5–21.3
Posterior margin	5.7	5.5–7.7	5.6–8.0	4.4–7.3
Height	2.3	2.1–3.6	2.2–3.3	2.0–3.0
**CAUDAL FIN**				
Dorsal lobe length	14.4	14.8–21.2	11.9–21.5	15.8–21.2
Ventral lobe length	10.6	8.6–16.6	8.2–15.8	10.8–15.2
Dorsal tip to notch	5.7	6.2–9.7	4.8–9.2	5.5–9.7
Notch depth	1.5	1.0–2.4	0.8–2.4	0.7–1.8
**DISTANCES BETWEEN FINS**				
Origin of pectoral to origin of pelvic fins	30.7	35.0–46.9	30.8–39.1	31.0–46.1
Base of pectoral to base of pelvic fins	25.0	29.5–39.2	24.2–33.2	26.6–35.6
Origin of pelvic to origin of caudal fin	11.7	8.3–14.5	8.8–12.3	9.5–13.4
Origin of 1st to origin of 2nd dorsal fin	14.8	16.0–23.4	13.2–18.6	14.0–20.4
Origin of 2nd dorsal to origin of caudal fin	10.4	7.0–11.5	6.5–9.4	6.9–11.1
**DENTITION** (tooth formula^‡^)				
Upper jaw	18–0–19	(17–24)–(0–1)–(17–24)	(15–25)–(0–1)–(15–24)	(18–24)–(0–1)–(18–24)
Lower jaw	25–0–27	(22–28)–(0–1)–(22–28)	(23–31)–(0–1)–(23–32)	(24–29)–(0–1)–(24–29)
**MERISTICS**				
Number of spiral valve turns	40	29–34	32–37	36–41

**Notes.**

† AO, anterior origin of the first dorsal fin; PO, posterior origin of the first dorsal fin.

‡ Tooth counts are reported as tooth files in the functional series (left–symphysis–right; L–S–R); replacement rows were not counted.

* Indicates that no comparative value was available in Yano, Stevens & Compagno (2004) for that character.

**Figure 4:**
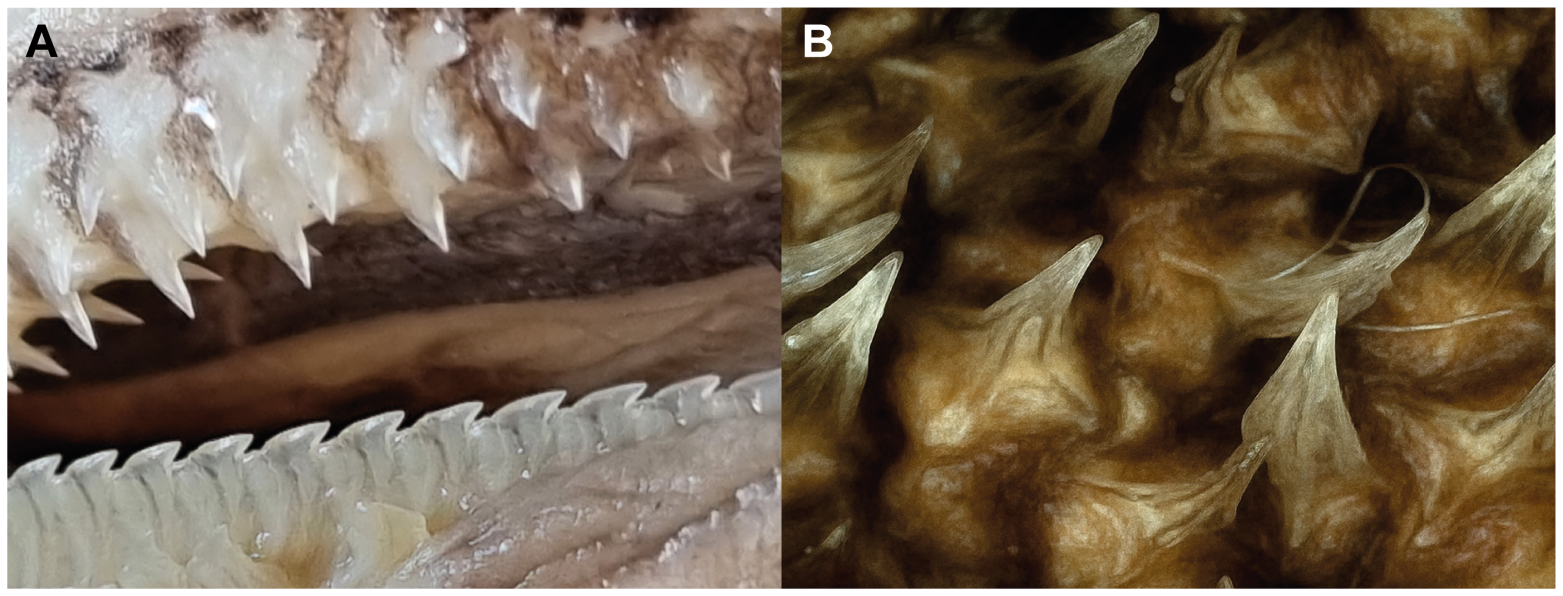
Details of dermal denticles and teeth of *S. antarcticus*. Diagnostic characteristics of *Somniosus antarcticus*. (A) Upper and lower jaws showing heterodont dentition, with lower teeth bearing short, strongly oblique cusps and high, narrow roots. (B) Dermal denticles with erect, narrow crowns, and hook-like cusps, giving the skin a rough spiny texture.

The dentition was heterodont. The upper jaw comprised 18–0–19 tooth files (total 37) with small lanceolate cusps, whereas the lower jaw comprised 25–0–27 tooth files (total 52) with short, strongly oblique cusps and very high, narrow roots (Fig. 4A). No symphyseal tooth files were observed in either jaw. Dermal denticles exhibited erect, narrow crowns with hook-like cusps, giving the skin a rough, spiny texture (Fig. 4B). These features, together with the external morphology, are consistent with the genus *Somniosus* and not *Rhinoscymnus*. The spiral valve contained 40 turns, which falls within the range reported for *S. antarcticus sensu* Yano, Stevens & Compagno (2004). Therefore, our morphological and meristic evidence is most consistent with *S. antarcticus* under the diagnostic framework of Yano, Stevens & Compagno (2004).

### Molecular analysis

A 639 bp fragment of the COI gene was obtained from both the gravid female and late-stage embryo. The two Chilean sequences were identical across the trimmed alignment, indicating a shared mitochondrial haplotype that was consistent with maternal inheritance. BLAST searches against GenBank returned *S. antarcticus* as the closest match, with 99.7–100% similarity to sequences from New Zealand and Antarctic waters (*p*-distance = 0.000–0.003). No other *Somniosus* species matched the Chilean haplotype as closely. The Chilean haplotype differed by two substitutions (*p*-distance = 0.00313) from the New Zealand record labelled *S. antarcticus* (JN312346) and was identical to an Antarctic record labelled *S. pacificus* (JN641139), indicating haplotype sharing across nominal labels.

**Figure 5:**
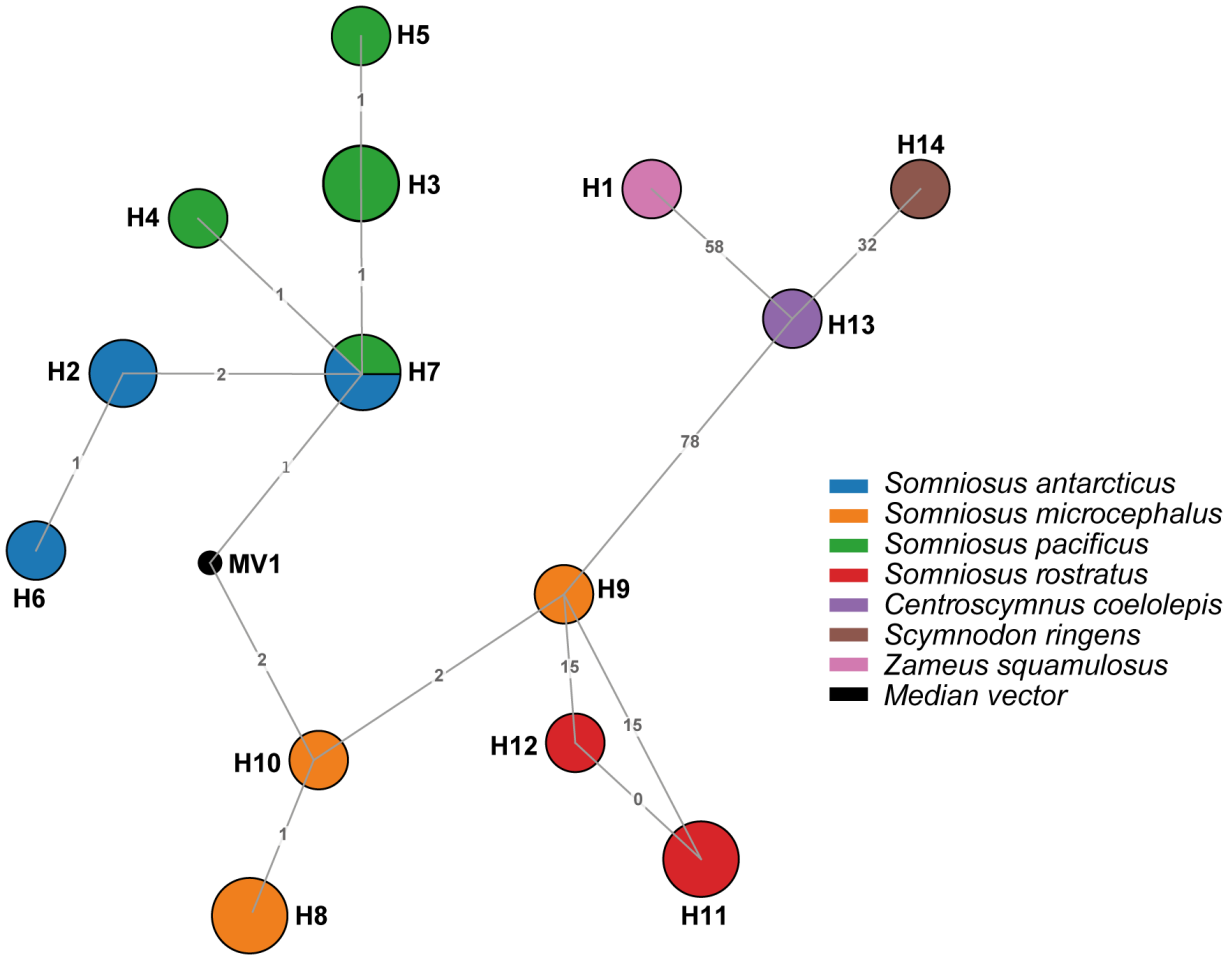
A median-joining (MJ) haplotype network based on a 639-bp fragment of the mitochondrial cytochrome c oxidase subunit I (COI) gene for *Sominosus* spp. Circle size is proportional to haplotype frequency. Colors indicate the nominal species label, as reported in GenBank. Hatch marks on branches represent mutational steps; small filled nodes represent inferred median vectors.

The comparative dataset comprised 27 sequences spanning four *Somniosus* taxa (*S. microcephalus*, *S. pacificus*, *S. antarcticus*, and *S. rostratus*), and three somniosid outgroups representing other genera within the family. Collapsing of identical sequences yielded 14 unique haplotypes. The female and embryo shared the same Chilean haplotype, identified as H2, in the median-joining network (Fig. 5). The median-joining network (epsilon = 0) recovered a shallow haplotype structure within the large-bodied *Somniosus* complex and highlighted a single haplotype shared between nominal *S. pacificus* and *S. antarcticus* (the Chilean haplotype), whereas *S. rostratus* formed a distinct haplotype group separated from the large-bodied complex by substantially larger mutational distances (Fig. 5).

**Figure 6:**
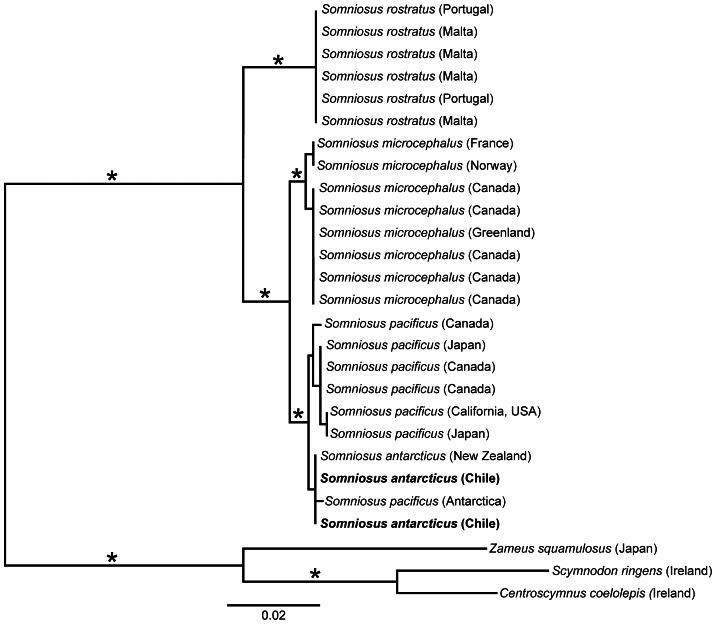
Distance-based neighbor-joining clustering visualization for *Somniosus* species complex derived from Kimura two-parameter (K2P) distances for the COI alignment used in haplotype analyses. Bootstrap support (1,000 pseudoreplicates) at branch nodes is shown (with an *) when >80%. Tip labels include GenBank accession numbers and nominal species assignments, as reported in GenBank (Table S1). Chilean specimens are indicated in bold. The three topologies were rooted using representative somniosid genera outside *Somniosus* as outgroups (Family Somniosidae).

The genetic distance summaries were consistent with the haplotype network (Table 2; Table S2). Within-group divergence was greatest in *S. microcephalus*, whereas nominal *S. pacificus* and *S. antarcticus* showed comparatively low internal variations. The divergence between *S. pacificus* and *S. antarcticus* also remained low, and the nearest-neighbor distance between these nominal labels was zero, reflecting haplotype sharing between the Chilean specimens and an Antarctic record labelled as *S. pacificus*. In contrast, *S. rostratus* and the somniosid outgroups were more divergent from the large-bodied *Somniosus* complex, supporting the separation of *Rhinoscymnus* and other somniosid lineages at this locus. A distance-based neighbor-joining reconstruction provided a complementary clustering visualization of these patterns (Fig. 6), recovering the same broad groupings and a shallow structure within the large-bodied *Somniosus* complex. Within this complex, two closely related clusters were recovered: one composed mainly of sequences labelled *S. pacificus* and another containing the Chilean haplotype together with sequences labelled *S. antarcticus*. However, one Antarctic sequence labelled *S. pacificus* clustered with the latter group, so nominal *S. pacificus* and *S. antarcticus* were only partially separated in the COI dataset. This pattern is consistent with the haplotype network and distance summaries, which showed low inter-label divergence and haplotype sharing among southern records.

**Table 2:** COI genetic distances for *Somniosus* spp. Within-group COI genetic distances and nearest-neighbor distances (NND) for *Somniosus* taxa included in the comparative dataset. Distances were calculated from a 639 bp alignment and are reported as mean ± SD, with minimum–maximum values in parentheses. NND represents the minimum intergroup distance between any sequence in the focal taxon/lineage and any sequence outside that taxon/lineage.

**Taxon/lineage**	***n*** **sequences**	**Pairwise comparisons**	***p*** **-distance, mean ± SD (min–max)**	**K2P distance, mean ± SD (min–max)**	**NND** **(p-distance)**	**NND (K2P)**
*S. microcephalus*	8	28	0.0050 ± 0.0042 (0.0000–0.0110)	0.0051 ± 0.0042 (0.0000–0.0110)	0.0031	0.0031
*S. pacificus*	7	21	0.0016 ± 0.0014 (0.0000–0.0047)	0.0016 ± 0.0014 (0.0000–0.0047)	0.0000	0.0000
*S. antarcticus*	3	3	0.0021 ± 0.0018 (0.0000–0.0031)	0.0021 ± 0.0018 (0.0000–0.0031)	0.0000	0.0000
Southern complex (*S. pacificus* + *S. antarcticus*)	10	45	0.0021 ± 0.0016 (0.0000–0.0063)	0.0021 ± 0.0016 (0.0000–0.0063)	0.0031	0.0031

## Discussion

Our integrative morphological and molecular evidence documents the occurrence of a large-bodied *Somniosus* off northern Chile and provides a curated record from a region where sleeper shark records have remained scarce and are uncertain taxonomically. Previous records of large-bodied *Somniosus* from Chilean waters included specimens reported from approximately 33°S and 40°S (Pequeño, Lamilla & Crovetto, 1991; Brito, 2004); however, these records predated the revision by Yano, Stevens & Compagno (2004) and lacked both genetic data and internal meristic information, which are now regarded as important for species identification. In this context, the present record strengthens the evidence that the large-bodied *Somniosus* occurring in Chilean waters belongs to the Southern Hemisphere lineage, traditionally referred to as *S. antarcticus sensu* Yano, Stevens & Compagno (2004).

A particularly important aspect of this record is the reproductive context. The small male was not captured independently but was aborted during handling, while the gravid female was being brought onboard. The direct observation of capture-induced abortion confirmed the sexual maturity of the female, representing a significant contribution to the reproductive biology of this genus. The estimated size of the gravid female appears small relative to previously published maturity estimates for the genus (approximately 300 to 400 cm TL, Finucci, 2018); however, this comparison should be interpreted cautiously because the estimate was derived from partial remains rather than from a complete specimen.

The Chilean embryo exhibits a combination of characters consistent with *Somniosus* and not *Rhinoscymnus*. These include hook-shaped dermal denticles, dentition, and external morphology compatible with a large-bodied sleeper shark, and a spiral valve count that clearly exceeds the range reported for small-bodied somniosids. Within the large-bodied *Somniosus* complex, the spiral valve count provides the strongest meristic support for consistency with *S. antarcticus*, under the framework of Yano, Stevens & Compagno (2004). Although several morphometric proportions overlap among large-bodied nominal species and may vary with ontogeny, our morphological and meristic evidence is most consistent with *S. antarcticus*, as currently understood under the diagnostic framework proposed by Yano, Stevens & Compagno (2004) and Yano, Stevens & Compagno (2007).

Our molecular results are compatible with this interpretation, but they also reinforce that COI does not fully resolve species boundaries within the *pacificus/antarcticus* complex (Timm et al., 2023). In the haplotype network, the Chilean haplotype was identical to an Antarctic sequence labelled *S. pacificus* and differed by only two substitutions from a New Zealand sequence labelled *S. antarcticus*. Accordingly, the divergence between nominal *S. pacificus* and *S. antarcticus* remained low, and the nearest neighbor distance between these taxa was zero. These patterns are consistent with previous mitochondrial studies showing shallow divergence and haplotype sharing among large-bodied *Somniosus* species in the Southern Hemisphere and adjacent regions (Benz et al., 2007; Murray et al., 2008; Christensen, 2022; Claassens et al., 2023).

The NJ reconstruction recovered two closely related clusters within the large-bodied *Somniosus* complex: one composed mainly of sequences labelled *S. pacificus* and another including the Chilean haplotype together with sequences labelled *S. antarcticus*. However, one Antarctic sequence labelled *S. pacificus* clustered with the latter group, indicating that nominal *S. pacificus* and *S. antarcticus* were not clearly separated by COI. This pattern is consistent with the network and distance analyses and supports the view that COI is informative for broad lineage placement but does not fully resolve species boundaries within this species complex (Murray et al., 2008; Christensen, 2022; Claassens et al., 2023).

In the present case, the Antarctic record labelled *S. pacificus* deserves particular caution because its taxonomic identity was assigned before the revised morphological framework of Yano, Stevens & Compagno (2004). This may represent a case of taxonomic uncertainty in the public record rather than true evidence of a well-differentiated Southern Hemisphere *S. pacificus*. Therefore, we interpret the Chilean specimens as belonging to the large-bodied Southern Hemisphere lineage that is most consistent with *S. antarcticus* sensu Yano, Stevens & Compagno (2004), while acknowledging that the precise boundary between *S. antarcticus* and *S. pacificus* remains unresolved with mitochondrial data alone. This interpretation is consistent with that of recent studies. Christensen (2022), based on complete mitogenomes, found no clear mitochondrial break between *S. pacificus* and *S. antarcticus*, and Timm et al. (2023) further questioned whether these nominal taxa represent distinct evolutionary lineages when evaluated using genomic data. Taken together, these studies suggest that the *pacificus/antarcticus* complex may reflect incomplete lineage sorting, shallow mitochondrial divergence, or an incompletely resolved taxonomic boundary. Under these circumstances, a pragmatic approach is warranted: large-bodied Southern Hemisphere *Somniosus* can be identified operationally as *S. antarcticus* sensu Yano, Stevens & Compagno (2004) based on morphology and internal meristics, while mitochondrial data are used primarily to document lineage affinity and to flag cases of taxonomic overlap or uncertainty.

Future studies integrating nuclear markers or genome-scale datasets with detailed morphological examination of vouchered specimens from the Southern Hemisphere, North Pacific, and Atlantic/Arctic regions are essential to determine whether *S. antarcticus* and *S. pacificus* represent distinct biological species or geographically structured components of a single, broader lineage. The Chilean specimens reported herein provide important data points from a previously undersampled region of the southeastern Pacific.

The Antofagasta record supports the occurrence of a large-bodied *Somniosus* consistent with *S. antarcticus* along the continental slope of northern Chile at approximately 22°S in deep waters associated with the Peru–Chile margin. This finding helps fill the geographic gap between Southern Ocean records and previous reports of large-bodied *Somniosus* spp. from central Chile (Pequeño, Lamilla & Crovetto, 1991; Brito, 2004; Yano, Stevens & Compagno, 2007; Ebert, Fowler & Compagno, 2021). More broadly, it supports the growing view that large-bodied *Somniosus* lineages occupy a wider latitudinal range in deep waters than previously documented, including temperate and, at least, occasionally subtropical environments (Acero et al., 2018; Claassens et al., 2023).

The reproductive context of this record is noteworthy. Capture-induced abortion of a late-stage embryo indicates that gravid females occur on the northern Chilean slope and suggests that these habitats may form part of the reproductive range of the southern large-bodied lineage. However, a single event is insufficient to infer the presence of discrete pupping or nursery areas. Repeated records of gravid females, embryos, or neonates are required before northern Chile can be interpreted as a recurrent reproductive habitat.

Deep-sea chondrichthyans typically share life-history traits that make them highly vulnerable to fishery-induced mortality, including slow growth, delayed maturity, and low fecundity (Finucci et al., 2022). Large-bodied *Somniosus* sharks are expected to conform to this pattern, and even infrequent capture may have disproportionate demographic consequences (Yano, Stevens & Compagno, 2007; Finucci, 2018). Our record provides evidence that deep-sea longline fisheries operating off northern Chile interact with large-bodied *Somniosus*, even though such captures appear to be uncommon. This pattern is consistent with observations from the same artisanal fleet targeting Patagonian toothfish (*Dissostichus eleginoides*) in austral waters, where sleeper sharks reported as *Somniosus pacificus*, and possibly corresponding to *S. antarcticus* sensu Yano, Stevens & Compagno (2004), occurred as bycatch and, despite being represented by only two individuals, contributed nearly 40% of the total deep-sea chondrichthyan biomass recorded in the study area (Reyes & Torres-Florez, 2009).

Bycatch monitoring programs that systematically document elasmobranch catches, record sex and length whenever possible, secure tissue samples such as fin clips, and retain diagnostic materials (*e.g.*, jaws and spiral valves) across both northern and southern fleets are essential to improve occurrence data and distinguish *Somniosus* species. Whenever possible, live individuals should be released into the wild, in line with the current recommendations of the National Fishery Authority for industrial longline and gillnet fisheries (Bustamante, Acuña & Vargas-Caro, 2024).

On a broader scale, taxonomic uncertainty should not prevent fishery monitoring or conservation actions. Until genome-scale analyses clarify the status of *S. antarcticus* and *S. pacificus*, fishery assessments may need to track these taxa pragmatically as a large-bodied *Somniosus* complex while still recording morphological evidence that supports operational identifications. The morphometric, meristic, and COI data presented here contribute to this framework and provide a useful regional reference for future studies in the southeastern Pacific.

## Supplemental Information

10.7717/peerj.21381/supp-1 Supplemental Information 1 Supplementary Tables

10.7717/peerj.21381/supp-2 Supplemental Information 2 Comparative context of morphometric and meristic data

10.7717/peerj.21381/supp-3 Supplemental Information 3 Novel genetic sequences from *Somniosus antarcticus* The raw sequences of the COI gene from specimens collected in northern Chile.

## Additional Information and Declarations

### Field Study Permissions

The following information was supplied relating to field study approvals (i.e., approving body and any reference numbers): All work was conducted under special research permits granted by the Chilean Subsecretaría de Pesca y Acuicultura (E-PINV-2021-598).

### Data Availability

The following information was supplied regarding data availability: The raw data and sequences are available in the Supplemental Files. The sequences are available at Genbank: PX559966 –PX559967.
