# Supplementary material for: Morphological and molecular evidence of the Antarctic sleeper shark Somniosus antarcticus (Somniosidae) in northern Chile
Source: PeerJ. 2026 Jun 26;14:e21381. doi: 10.7717/peerj.21381 (PMC13313001; doi:10.7717/peerj.21381)
Supplement: Supplemental Information 1 [file peerj-14-21381-s001.docx]

**Morphological and molecular evidence of the Antarctic sleeper shark *Somniosus antarcticus* (Somniosidae) in northern Chile**

Angel Mancilla^1,2^, Leandro Brizuela^1,2^, Krishna Tapia^1,2^, Carolina Vargas-Caro^1,2^, Carlos Bustamante^1,2^

**Supplementary Material**

**Table S1:** Reference COI sequences used for comparative analyses of Somniosidae. For each record, the GenBank accession number, nominal species identification as reported in the original source, sampling locality, and reference are indicated. The species sequences used as outgroups in the analysis are indicated with an asterisk (*).

| **Species** | **GenBank ID** | **Location** | **Reference** |
| --- | --- | --- | --- |
| *Somniosus microcephalus* | EU148328 | Norway | Byrkjedal et al. (2007) |
| *Somniosus microcephalus* | KY513709 | Greenland | Santaquiteria et al. (2017) |
| *Somniosus microcephalus* | GU130677 | France | Straube et a. (2009) |
| *Somniosus microcephalus* | KC015919 | Canada (Nova Scotia) | McCusker et al. (203) |
| *Somniosus microcephalus* | KC015920 | Canada (Nova Scotia) | McCusker et al. (203) |
| *Somniosus microcephalus* | KC015921 | Canada (Nova Scotia) | McCusker et al. (203) |
| *Somniosus microcephalus* | KC015922 | Canada (Nunavut) | McCusker et al. (203) |
| *Somniosus microcephalus* | KC015923 | Canada (Nova Scotia) | McCusker et al. (203) |
| *Somniosus rostratus* | KJ083255 | Portugal | Moura et al. (2015) |
| *Somniosus rostratus* | OX638082 | Portugal | Cordone et al. (2023) |
| *Somniosus rostratus* | KY909543 | Malta | Vella et a. (2017) |
| *Somniosus rostratus* | KY909544 | Malta | Vella et a. (2017) |
| *Somniosus rostratus* | KY909545 | Malta | Vella et a. (2017) |
| *Somniosus rostratus* | KY909546 | Malta | Vella et a. (2017) |
| *Somniosus pacificus* | FJ165366 | Canada (British Columbia) | Wong et a. (2009) |
| *Somniosus pacificus* | FJ519585 | Canada (British Columbia) | Wong et a. (2009) |
| *Somniosus pacificus* | FJ519586 | Canada (British Columbia) | Wong et a. (2009) |
| *Somniosus pacificus* | JF952860 | Japan | Zhang & Hanner (2011) |
| *Somniosus pacificus* | HQ010083 | USA (California) | Hastings & Burton (2008) |
| *Somniosus pacificus* | AB560492 | Japan | Tanaka et al. (2013) |
| *Somniosus pacificus* | JN641139 | Antarctica (South Georgia) | Smith et al. (2011) |
| *Somniosus antarcticus* | JN312346 | New Zealand | Stewart (2011) |
| *Somniosus antarcticus* | PX559966 | Chile | Present work |
| *Somniosus antarcticus* | PX559967 | Chile | Present work |
| **Centroscymnus coelolepis* | EU003883 | Ireland | Moura et al. (2008) |
| **Scymnodon ringens* | GU130697 | Ireland | Straube et al. (2010) |
| **Zameus squamulosus* | DQ108208 | Australia | Ward et al. (2005) |

**Table S2:** Between-group COI genetic distances among *Somniosus* taxa included in the comparative dataset. Distances were calculated from a 639 bp alignment and are reported as mean ± SD, with minimum–maximum values in parentheses, for both uncorrected *p*-distance and Kimura two-parameter (K2P) distance.

| **Group 1** | **Group 2** | ***n* comparisons** | ***p*-distance, mean ± SD (min–max)** | **K2P distance, mean ± SD (min–max)** |
| --- | --- | --- | --- | --- |
| *S. microcephalus* | *S. pacificus* | 56 | 0.0070 ± 0.0018 (0.0031–0.0110) | 0.0071 ± 0.0018 (0.0031–0.0110) |
| *S. microcephalus* | *S. antarcticus* | 24 | 0.0057 ± 0.0014 (0.0031–0.0078) | 0.0058 ± 0.0014 (0.0031–0.0079) |
| *S. pacificus* | *S. antarcticus* | 21 | 0.0026 ± 0.0017 (0.0000–0.0063) | 0.0026 ± 0.0017 (0.0000–0.0063) |
| *S. microcephalus* | Southern complex | 80 | 0.0067 ± 0.0018 (0.0031–0.0110) | 0.0067 ± 0.0018 (0.0031–0.0110) |

**References**

Byrkjedal, I., Willassen, E. & Hanner, R. (2007). *Barcoding deep sea fishes from the northern mid Atlantic*. Second International Barcode of Life Conference.

Hastings, P.A. & Burton, R.S. (2008). *Establishing a DNA Sequence Database for the Marine Fish Fauna of California*. Research Final Reports, University of California.

Cordone, A., Selci, M. & Giovannelli, D. (2020). *Shark microbiome analysis demonstrates unique microbial communities in two distinct Mediterranean Sea shark species*. University of Naples Federico II, Italy.

McCusker, M.R., Denti, D., Van Guelpen, L., Kenchington, E. & Bentzen, P. (2013). Barcoding Atlantic Canada's commonly encountered marine fishes. *Molecular Ecology Resources* 13, 177–188.

Moura, T., Silva, M.C., Figueiredo, I., Neves, A., Muñoz, P.D., Coelho, M.M. & Gordo, L.S. (2008). Molecular barcoding of north-east Atlantic deep-water sharks: species identification and application to fisheries management and conservation. *Marine & Freshwater Research* 59, 214–223.

Moura, T., Silva, M.C. & Figueiredo, I. (2015). Barcoding deep-water chondrichthyans from mainland Portugal. *Marine & Freshwater Research* 66, 508–517.

Santaquiteria, A., Nielsen, J., Klemetsen, T., Willassen, N.P. & Præbel, K. (2017). The complete mitochondrial genome of the long-lived Greenland shark (*Somniosus microcephalus*): characterization and phylogenetic position. Conservation Genetic Resourses 9, 351–355.

Smith, P.J., Steinke, D., Dettai, McMillan, P., Welsford, D., Stewart, A. & Ward, R.D. (2012). DNA barcodes and species identifications in Ross Sea and Southern Ocean fishes. *Polar Biology* 35, 1297–1310.

Straube, N., Iglésias, S.P., Sellos, D.Y., Kriwet, J. & Schliewen, U.K. (2010). Molecular phylogeny and node time estimation of bioluminescent Lantern Sharks (Elasmobranchii: Etmopteridae). *Molecular Phylogenetics and Evolution* 56(3), 905–917.

Steward, A., (2011). *Somniosus antarcticus*, International Barcode of Life., Centre for Biodiversity Genomics, Museum of New Zealand Te Papa Tongarewa, New Zealand.

Tanaka, K., Shiina, T., Tomita, T., Suzuki, S., Hosomichi, K., Sano, K., Doi, H., Kono, A., Komiyama, T., Inoko, H., Kulski, J.K. & Tanaka, S. (2013). Evolutionary Relations of Hexanchiformes Deep-Sea Sharks Elucidated by Whole Mitochondrial Genome Sequences. *BioMed Research International* 2013, 147064.

Vella, A., Vella, N. & Schembri, S. (2017). A molecular approach towards taxonomic identification of elasmobranch species from Maltese fisheries landings. *Marine Genomics* 36, 17–23

Ward, R.D., Zemlak, T.S., Innes, B.H., Last, P.R. & Hebert, P.D.N. (2005). DNA barcoding Australia’s fish species. *Philosophical Transactions of the Royal Society B: Biological Sciences* 360(1462), 1847–1857.

Wong, E.H.K., Shivji, M.S. & Hanner, R.H. (2009). Identifying sharks with DNA barcodes: assessing the utility of a nucleotide diagnostic approach. *Molecular Ecology Resources* 9, 243–256.

Zhang, J.B. & Hanner, R. (2011). DNA barcoding is a useful tool for the identification of marine fishes from Japan. *Biochemical Systematics and Ecology* 39(1), 31–42.
