# Supplementary material for: Morphological and molecular evidence of the Antarctic sleeper shark Somniosus antarcticus (Somniosidae) in northern Chile
Source: PeerJ. 2026 Jun 26;14:e21381. doi: 10.7717/peerj.21381 (PMC13313001; doi:10.7717/peerj.21381)
Supplement: Supplemental Information 3 — The raw sequences of the COI gene from specimens collected in northern Chile. [file peerj-14-21381-s003.docx]

>SAN_A360 Somniosus antarcticus Antofagasta (Chile)

TTTGGTGCCTGAGCAGGGATAGTAGGCACAGCCCTGAGTTTACTTATTCGAACAGAATTAAGCCAACCAGGAACACTTCTAGGAGATGATCAAATCTACAATGTTATTGTTACTGCTCACGCTTTCGTAATAATCTTTTTTATAGTAATGCCTGTAATAATTGGCGGGTTCGGAAATTGATTAGTCCCTCTAATAATTGGCGCACCCGACATAGCTTTCCCGCGAATAAATAACATAAGCTTTTGATTACTCCCCCCCTCTCTCCTACTACTTTTAGCCTCTGCCGGGGTTGAAGCAGGAGCCGGAACCGGCTGAACGGTCTATCCCCCCCTTGCAGGTAATATAGCCCACGCCGGCGCATCCGTAGACTTAGCCATCTTCTCACTCCACTTGGCTGGTATTTCATCAATTTTAGCCTCTGTTAACTTCATCACAACTATTATTAATATAAAACCACCTGCCATTTCTCAATATCAAACACCACTATTTGTCTGATCCATCCTTGTAACTACAGTCCTCCTACTCCTTTCCCTTCCTGTTCTTGCAGCTGCAATCACAATACTATTAACCGACCGTAATTTAAACACAACATTTTTTGACCCTGCTGGAGGAGGAGACCCAATTCTCTATCAACACCTA

>SAN_A244 Somniosus antarcticus Antofagasta (Chile)

TTTGGTGCCTGAGCAGGGATAGTAGGCACAGCCCTGAGTTTACTTATTCGAACAGAATTAAGCCAACCAGGAACACTTCTAGGAGATGATCAAATCTACAATGTTATTGTTACTGCTCACGCTTTCGTAATAATCTTTTTTATAGTAATGCCTGTAATAATTGGCGGGTTCGGAAATTGATTAGTCCCTCTAATAATTGGCGCACCCGACATAGCTTTCCCGCGAATAAATAACATAAGCTTTTGATTACTCCCCCCCTCTCTCCTACTACTTTTAGCCTCTGCCGGGGTTGAAGCAGGAGCCGGAACCGGCTGAACGGTCTATCCCCCCCTTGCAGGTAATATAGCCCACGCCGGCGCATCCGTAGACTTAGCCATCTTCTCACTCCACTTGGCTGGTATTTCATCAATTTTAGCCTCTGTTAACTTCATCACAACTATTATTAATATAAAACCACCTGCCATTTCTCAATATCAAACACCACTATTTGTCTGATCCATCCTTGTAACTACAGTCCTCCTACTCCTTTCCCTTCCTGTTCTTGCAGCTGCAATCACAATACTATTAACCGACCGTAATTTAAACACAACATTTTTTGACCCTGCTGGAGGAGGAGACCCAATTCTCTATCAACACCTA
